# Telangiectatic Mastocytosis: If It Is Not Mastocytosis, What Is It? Comment on Brockow et al. Challenges in the Diagnosis of Cutaneous Mastocytosis. *Diagnostics* 2024, *14*, 161

**DOI:** 10.3390/diagnostics15111370

**Published:** 2025-05-29

**Authors:** Francisco Urbina, Alicia Benavides

**Affiliations:** 1 Independent Researcher, Santiago de Chile 7580258, Chile; 2 Laboratorio de Histopatología, Clínica Redsalud Providencia, Santiago de Chile 7500995, Chile

**Keywords:** mast cells, mastocytosis, telangiectasias, telangiectatic mastocytosis, telangiectasia macularis eruptiva perstans, TMEP, mast cell activation syndrome

We read with great interest the recent review of Brockow et al. [1] about the challenges in the diagnosis of cutaneous mastocytosis, a topic in which we are particularly interested. As the authors have stated, the term cutaneous mastocytosis is ambiguous, and the less typical manifestations with hardly visible skin lesions can make diagnosis difficult (“occult mastocytosis”) [1]. This can leave some cases in no man’s land, without a definitive diagnosis and with no guidelines to follow. In addition, on histopathological grounds there is not a clear cutoff in the number of mast cells counted in skin biopsies to aid in a definitive diagnosis, something that has become increasingly stricter over the years. Different counting methods have been used, including per high-power view (×400), per mm^2^, per mm^3^, and per vascular unit, without being homologous or comparable between different publications. When one of us became involved in the subject, almost 30 years ago, there was no publication that defined at what level the mast cell count was considered abnormal; among those who ventured to give figures, up to 10 mast cells per high-power view was considered normal [2], or even less. On the other hand, count results vary depending on the biopsied area (face and acral extremities could present more mast cells than the trunk) and also according to the personal criteria of each pathologist (only nucleated mast cells, not including counts around adnexal structures or dermal nerves, etc.) [3]. It has been suggested that a figure up to 75 mast cells/mm^2^ can be seen in normal skin, more than 250 most likely confirms mastocytosis, and between 75 and 250 is a range in which mastocytosis should be considered [3]. Others conclude around 40 mast cells/mm^2^ in normal skin [4], with 4- to 8-fold increase in the lesional dermis of patients with cutaneous mastocytosis, and about 2- to 3-fold increase in the skin of patients with inflammatory diseases.

Our purpose is to refer to a condition that has not received enough attention, in which the predominant sign is the presence of multiple telangiectasias associated with various systemic symptoms attributable to the release of mast cell mediators. We are not referring to telangiectasia macularis eruptiva perstans (TMEP), but rather to lesions made up only of telangiectasias, without a reddish-brown macular background.

In 1998, one of us described a case series of eight patients [5] with prominent telangiectasias distributed in different patterns that, when skin-biopsied, showed abnormal elevations in mast cell count in the superficial/mid-dermis or perivascular, ranging from 15 to 30 per high-power view (×400, Giemsa stain at those years). The basal layer was not hyperpigmented. Two women with abundant telangiectasias but without systemic symptoms served as controls, showing a mast cell count ranging from 0 to 4 per high-power view. All cases presented diverse symptomatology, including epistaxis, spontaneous tachycardia, spontaneous ecchymosis, nausea, abdominal pain, headaches, facial flushing and itching of the skin with sun exposure, among others, with variable associations between them. Five out of eight had dermographism. Most of these patients— and other similar cases subsequently attended —were often resigned and accustomed to their symptoms, having consulted various specialists including neurologist (headaches), gastroenterologist (diarrhea, abdominal pain, nausea), cardiologist (tachycardia), otorhinolaryngologist (epistaxis) and even psychiatrist (loss of consciousness classified as hysteria, depression, etc.). Depending on the symptoms, some had undergone studies with brain tomography scan, echocardiogram, electrocardiogram, gastric endoscopy, etc., always with normal results. Most of them showed improvement of their symptomatology with the use of antihistamines or oral disodium cromoglycate, which even produced relief of extradigestive symptoms in some cases. Systemic involvement was ruled out in all of them by abdominal ultrasound, bone scintigraphy and routine blood tests. The study with serum tryptase was not carried out at that time. It was concluded that these forms of “telangiectatic mastocytosis” were not rare but unrecognized, with an apparently benign course, and requiring a high index of suspicious for diagnosis.

In two of those eight patients initially seen, who consulted one of us (F.U.) many years later for other unrelated reasons, their symptoms and signs of mastocytosis had disappeared. Notably, one of them, a young male teenager whose main problem was burning erythema when touching or being touched on his ears (having to sleep with moist cloths on hand to mitigate discomfort during sleep), presented with a total disappearance of his telangiectasias in both ears, and without redness or discomfort when touched. Only one similar case has been reported, with recurrent inflammatory episodes in one ear and a delayed diagnosis until stains for mast cells were performed, detecting an average of 18 mast cells per high-power field (range 10–29 ×400) while healthy skin showed 0–4 mast cells ×400 [6].

As an example, we would like to comment on two patients with similar characteristics and a challenging diagnosis that we initially saw three months ago and whose conditions are currently under control.

**Case 1.** An 11-year-old boy consulted for a unilateral eruption on his right arm and forearm that had spontaneously appeared without a clear cause after a soccer game, two days before, with no history of trauma not even scratches on the area. One of his grandparents had died from some type of leukemia, and his parents were worried. He was apparently healthy, had no history of atopy or other diseases, and was not taking any medication.

Upon examination, he presented isolated and monomorphic macular violaceous lesions with an ecchymotic appearance that did not blanch under pressure, was were? distributed at the external side of his upper right extremity and was were? accompanied by a slight burning sensation (Figure 1). The lesions had an artifactual appearance with a linear distribution. There were no eczematous features on the surface of the skin, which appeared soft and smooth, apart from more external, mild keratosis pilaris. On the rest of clinical examination, he had multiple telangiectasias on his trunk, especially noticeable on the upper back and a marble-like skin on his hips, arms (Figure 2) and thighs. He also presented dermographism when scraped on the trunk.

When questioned directly, he reported having had two other previous episodes of ecchymotic-looking lesions on his chest—of the size of the palm of the hand—without associated trauma, which spontaneously remitted in two weeks, and without having gone through the color changes typical of a bruise. On both occasions, he noticed them when he got up in the morning. In addition, and also during this year, he had had multiple episodes of epistaxis that finally disappeared with electrocautery performed by an otorhinolaryngologist. He also reported an occasional urgency to defecate and episodes of spontaneous tachycardia without apparent cause while being at rest. When this happened, he measured the rate of his heartbeats with his watch, once reaching 161 per minute. Also during this year, he has had concentration problems, with poor performance in some school subjects. A series of blood tests were requested and, if normal, a skin biopsy would be scheduled for the following week.

The results of the analysis were normal or negative, including complete blood cell count, platelet count, erythrocyte sedimentation rate, prothrombin, partial thromboplastin, antinuclear antibodies, anticardiolipin antibodies, liver function test and urine profile. Serum tryptase level was within normal range (6.14 µg/L). When he was seen six days after to undergo biopsy, the arm lesions had completely disappeared without leaving any sequelae.

Two skin samples were taken from the upper and lower back, both showing slight, basal cell hyperpigmentation and an inflammatory infiltrate in the superficial dermis (Figure 3). Immunohistochemical stains with tryptase and CD117 showed that the infiltrate included mast cells (Figure 4), mostly with perivascular distribution. Mast cell count in six samples ranged from 11 to 12 mast cells per high-power view (CD117 and tryptase, respectively). The count per mm^2^ showed 34 mast cells with tryptase and 48 with CD117. C-kitD816V mutation analysis was not available. The bone scintigram did not detect any alterations and an abdominal ultrasound ruled out visceromegaly and only showed 10 mesenteric lymph nodes of non-specific appearance. He is currently under control and receiving oral levocetirizine 5 mg at night.

He was recently evaluated by a pediatric neurologist and diagnosed with combined attention deficit disorder. General guidelines were provided to his teachers to assist with behavioral and academic management. He was also instructed to start pharmacological treatment with methylphenidate 36 mg/day and aripiprazole 2.5 mg every 12 h.

**Case 2.** A 38-year-old woman consulted for long-standing pruritus and dermographism, which she had recently controlled with fexofenadine 180 mg at night with some improvement. Questioned directly about other complaints, she also mentioned frequent diarrhea that had been classified as irritable bowel syndrome. In recent months, diarrhea occurred with a frequency of 3 to 4 days a week, without having detected any precipitating factor. She has been taking omeprazole for many years for gastric heartburn, with normal endoscopic study. She also reported episodes of spontaneous tachycardia and recurring headaches. The latter appeared especially in cold weather; thus, she goes to bed with a sleeping cap during winter to prevent them. The episodes of tachycardia occurred at rest, especially after a hard day at work, in the form of a few successive episodes of short duration. Upon examination, she had multiple telangiectasias on her back and neckline (Figure 5), with marked dermographism. A serum tryptase test was requested and a skin biopsy was scheduled. She was told to add rupatadine during the day, that made her feel much better initially. Her tryptase value was within normal range (5.37 µg/L).

A skin biopsy showed basal cell hyperpigmentation and a chronic superficial perivascular inflammatory infiltrate that also affected the periphery of the hair follicles (Figure 6). Tryptase stain reached figures of up to 29 mast cells per high-power view (67/mm^2^), and CD117 up to 46 mast cells per high-power view (92/mm^2^), mostly surrounding the hair infundibulum (Figure 7), although some interstitial mast cells were also observed (Figure 8). PAS stain was negative for yeasts or fungal hyphae. Abdominal ultrasound study did not detect any anomalies. The bone scintigram detected mild non-specific osteoblastic lesions in the L5, S1 and sacroiliac joints (pending further study).

None of the previously studied patients—and both reported here—have symptoms that correspond to the differential diagnoses of mastocytosis, as proposed by Brockow et al. in their Table [1], such as urticaria, atopic dermatitis and many others which are impossible to capture based on their characteristic features and the very different clinical manifestations in our patients. All these cases simply consist of patients with diverse symptoms and variable associations between them, who upon examination present noticeable telangiectasias without any cause to explain them. They do not constitute rosacea, unilateral nevoid telangiectasia, Rendu–Osler–Weber syndrome, photoaging, or many other entities in which the main clinical finding are telangiectasias.

Nor do they correspond to the recently described mast cell activation syndrome (they may have some of its symptoms and respond to antihistamines, but they may lack elevated tryptase). How would they be classified then? Moreover, this ‘syndrome’ overlaps with too many conditions, without benefit for either the clinician or the patient [7] and almost all clinical medicine was included in it, even mastocytosis itself. It has no practical use and confuses and even complicates things for clinicians. Its most typical signs and symptoms are flushing and hypotension, followed by nasal congestion, and nasal pruritus, headache and diarrhea, urticaria and throat swelling, angioedema and wheezing [8], symptomatology far from that observed in our cases.

The greatest similarity of these cases is with the so-called TMEP, although with a complete lack of a brown macular background. Incredibly, this condition has been eliminated in the latest classifications of mastocytosis, because it could simply represent a highly vascularized form of maculopapular CM with dilated vessels or because of overlap with urticaria pigmentosa, even if the predominant clinical feature is telangiectasia [9]; but then, it would be mastocytosis! Although some publications about TMEP can be questioned, there are several in which its association with extracutaneous symptoms and even its evolution to systemic forms have been clearly demonstrated [10]. Telangiectasias are believed to occur due to persistent vasodilation induced by chronic release of mast cell mediators, particularly histamine, angiogenic factors and heparin, with the latter probably also involved in its hemorrhagic manifestations (epistaxis and ecchymosis). Familial TMEP has also been described.

A validation of dermatopathological criteria for the diagnosis of cutaneous mastocytosis has been recently proposed [11]. Although all the studied cases included unequivocal confirmed mastocytosis (maculopapular, mastocytosis in the skin, mastocytoma, and systemic mastocytosis), none were based on the prominent finding of telangiectasias. Major criteria for diagnosis were the presence of the KIT-D816V mutation, or the finding of more than 139 mast cells/mm^2^ (equivalent to about 27 mast cells per high-power view in the opinion of the authors), the latter a finding that adds 3 points. Minor criteria were a mast cell density equal or more than 12 mast cells per high-power view (adds 2 points), equal or more than 7 mast cells per high-power view (adds 1 point), intermedial or strong basal cell hyperpigmentation (adds 1 point), interstitial mast cell distribution (adds 1 point), and mast cell clusters equal to or more than 3 (adds 1 point). A final score of 4–6 points make diagnosis highly likely, with a specificity of 97.2% and sensitivity of 90.6%.

The two herein reported cases showed mast cell infiltrates—although mild as described in TMEP—and normal serum tryptase values. Would greater infiltrates have been detected with more biopsies from other sites (or for comparison)? Should bone marrow biopsies be indicated in both cases described? What conclusion can one draw if in most of the analyzed fields the mast cell count is 11 or 12, but in only one it increases to 21? Or just 27? Or shows some interstitial cells, or only two mast cell clusters? How many fields should be analyzed? Is it so essential to exceed a threshold figure for the diagnosis, even when all clinical symptoms clearly point to a form of mastocytosis? Otherwise, how should guidelines encompass their varied symptoms and lesions? (All of them are clearly attributable to mast cell mediators.) In this regard, anyone can occasionally have a headache or an episode of diarrhea, but not persistently over time, along with epistaxis and spontaneous tachycardia (or/and other complaints). Should these unrelated symptoms/signs—and others, including telangiectasia—be scored for mastocytosis if there are multiple and they persist for years without a cause or disease that explains them as a whole? Regarding our first case, all of his symptoms and signs started in the same year: epistaxis, tachycardia, episodes of cutaneous lesions and diminished school performance. Recently, in a systematic review about cognitive, neuropsychiatric and neurological alterations in mastocytosis, it was found that learning difficulties may be detected in approximately 13% of cases of pediatric mastocytosis, irritability in 6%, attention deficit hyperactivity in 12% and autism in 10%, contrasting with much lower figures in the general population [12].

As can be seen, different criteria about mast cell count have been raised by several authors, but none considers that the clinical findings are what set the tone and that in the end they should be prioritized for a final diagnosis, regardless of an absolutely fixed or required figure in mast cell count, highly variable for multiple causes. Serum tryptase, histamine and other measurements can also present variable results (or be expensive, or unavailable routinely worldwide), sometimes within normal ranges despite having proved to be mastocytosis. The finding of elevated periadnexal figures of mast cells in our second case is clearly abnormal if compared with normal skin and with other cases of mastocytosis; perhaps this reason for its exclusion in the counts should be redefined or at least be more studied. Apart from the figures given in one study [11], describing periadnexal mast cells in frequencies from 10% in control cases with urticaria to 42.9% in control cases with pruritus (compared with 6.3% in maculopapular cutaneous mastocytosis), we have not found a defined number of periadnexal mast cells in normal skin to have as a basis. That same study draws attention to the discrepancy in mast cell count figures according to the different stains used: while in cases of proven mastocytosis, the median mast cell density was almost equal with trypsin and CD117 at any cutaneous level (overall, subepidermal, intermediate and lower dermis), in the control group, those values were notoriously dissimilar, with a median four or more times higher with the use of tryptase than with CD117.

In conclusion, these patients will continue to carry their symptoms and be hopelessly resigned to their condition, frustratedly consulting specialist to specialist without obtaining results or an accurate diagnosis, unless some simple and affordable definition is standardized, including for TMEP—which if it exists, it exists.

## Figures and Tables

**Figure 1:**
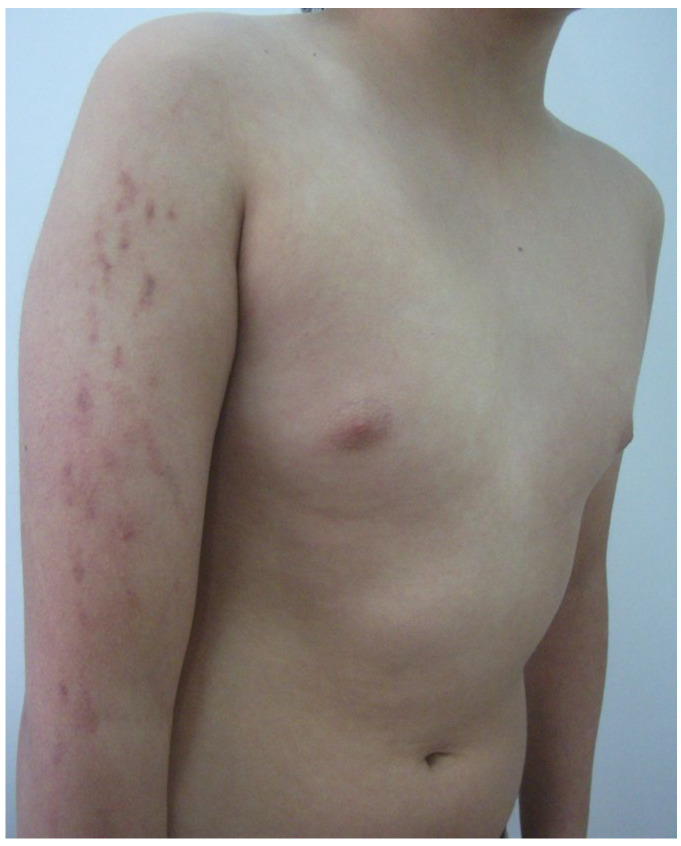
Violaceous macular lesions with ecchymotic appearance, especially noticeable and darker in the higher ones near the shoulder.

**Figure 2:**
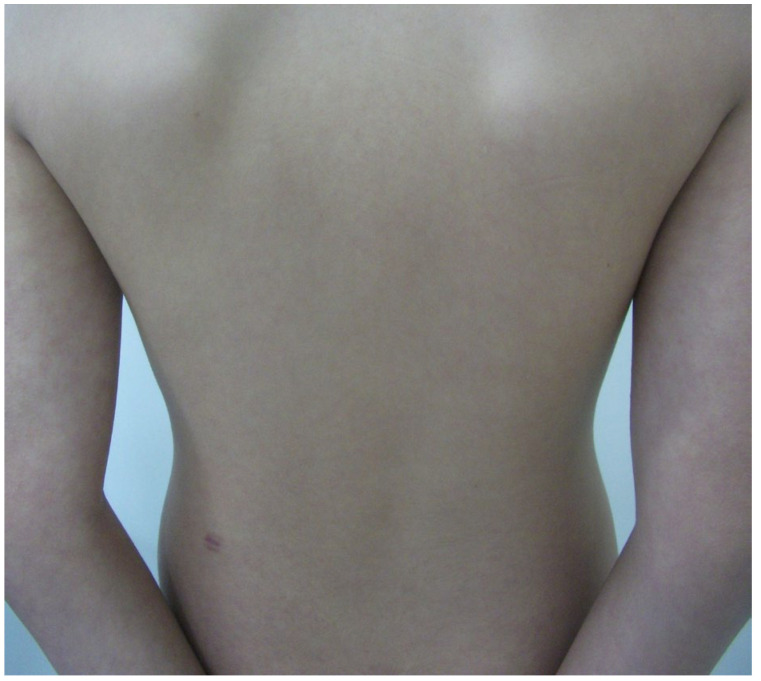
Telangiectasias on back and marble-like skin on arms and forearms. One biopsy scar on left lower back.

**Figure 3:**
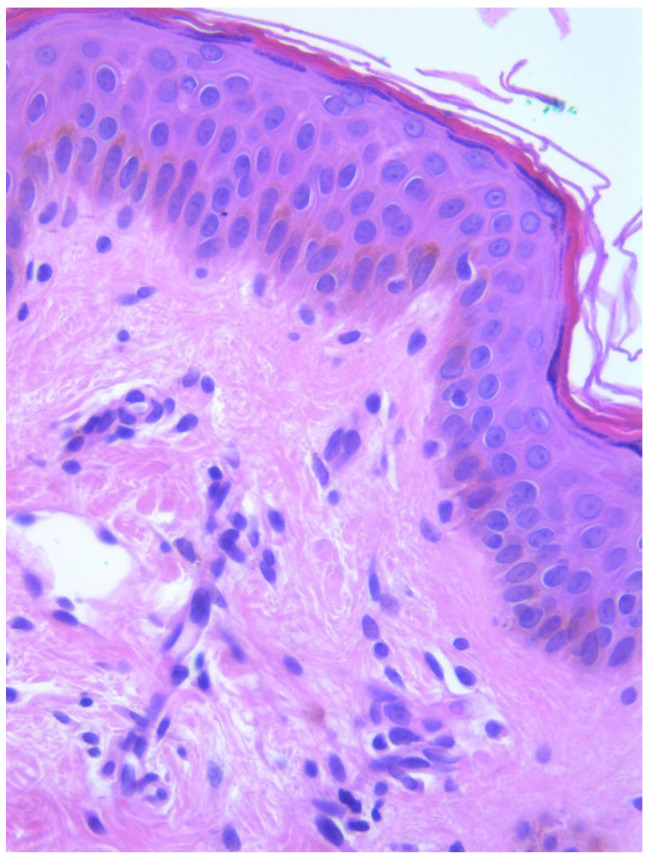
Slight basal cell hyperpigmentation and superficial inflammatory infiltrate (He, ×100).

**Figure 4:**
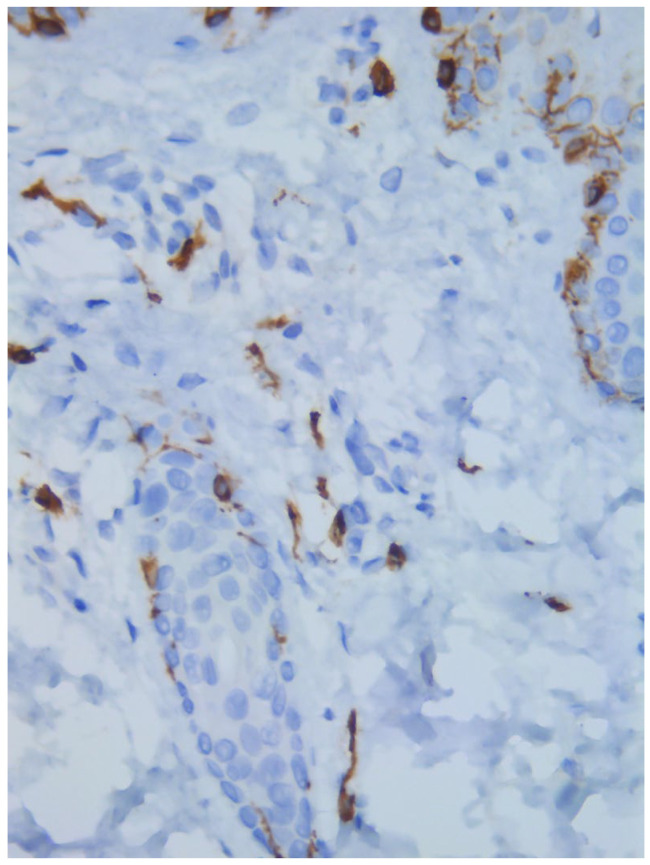
Mast cell infiltration (CD117, ×400).

**Figure 5:**
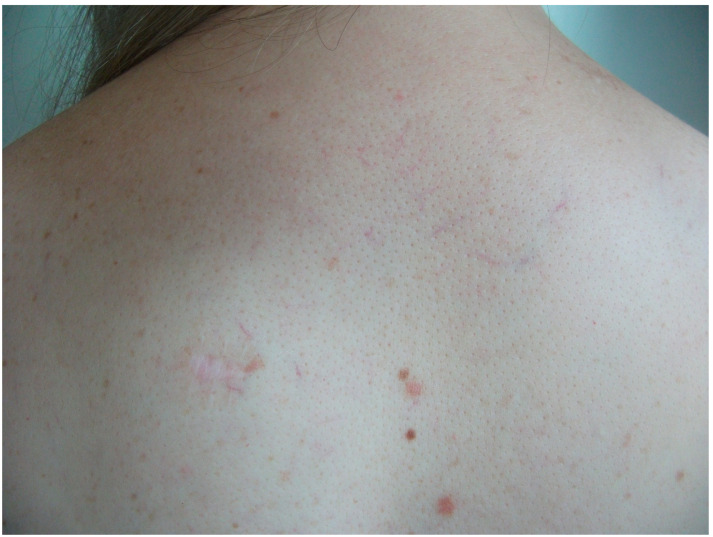
Numerous telangiectatic vessels on the upper back. Below left is the biopsy scar.

**Figure 6:**
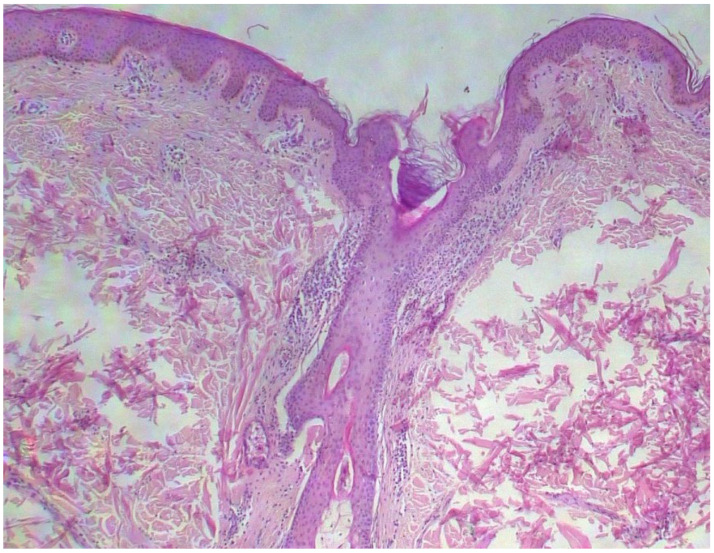
Basal cell pigmentation and superficial dermal inflammatory infiltrate, mostly surrounding the pilosebaceous infundibulum (HE ×40).

**Figure 7:**
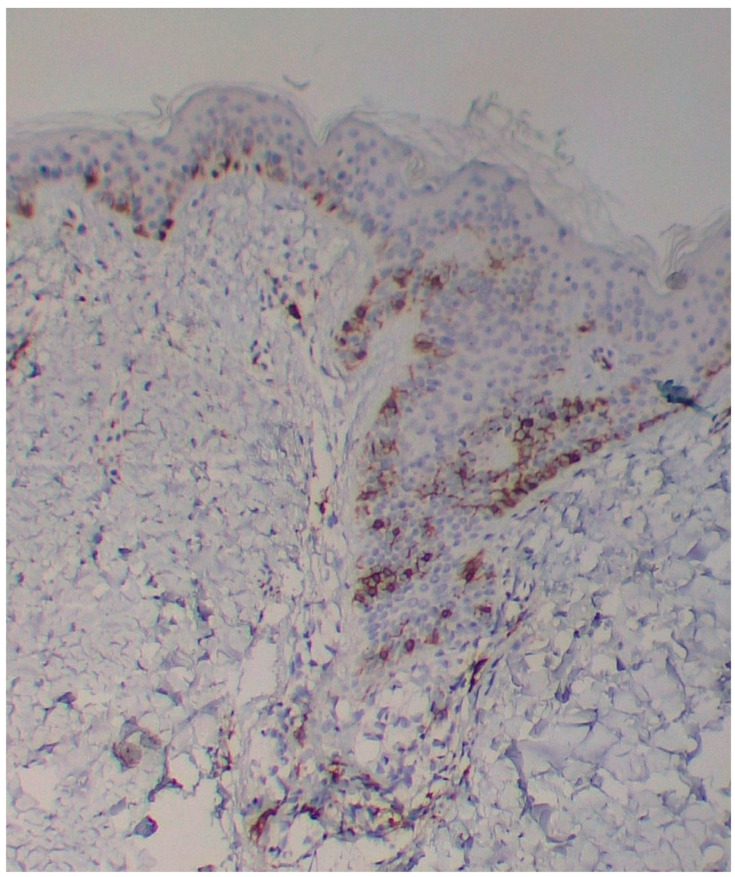
Mast cell infiltration in superficial dermis and around a pilosebaceous follicle (CD117 ×100).

**Figure 8:**
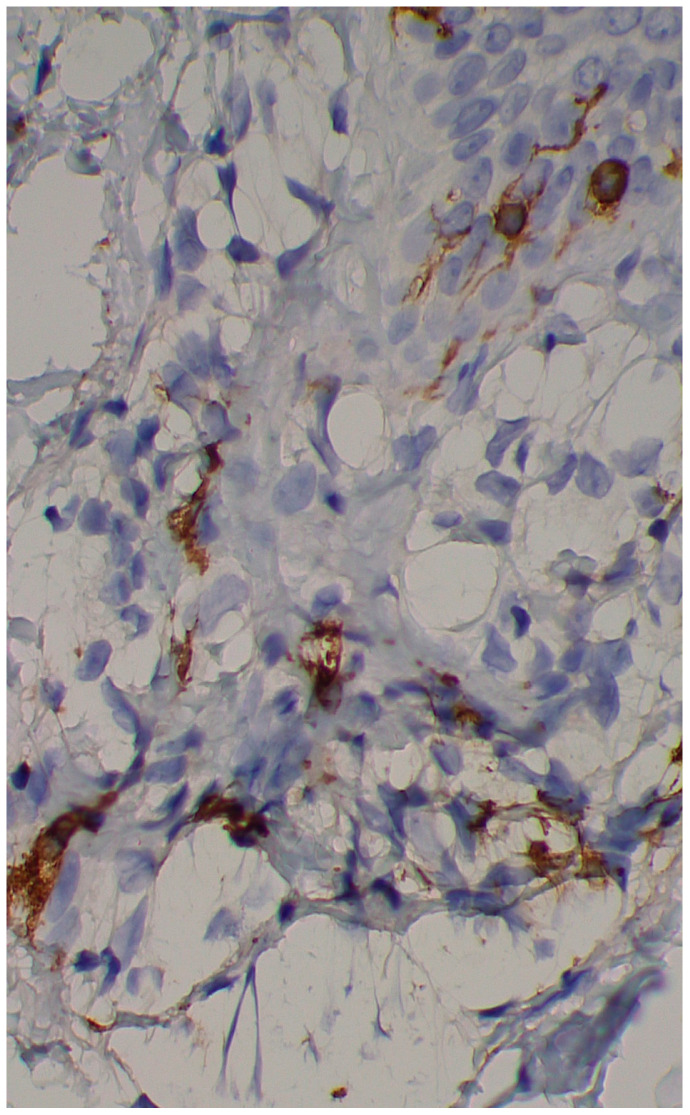
Mast cell infiltration (CD117 ×400).
